# Recent Progress in Gd-Containing Materials for Neutron Shielding Applications: A Review

**DOI:** 10.3390/ma16124305

**Published:** 2023-06-10

**Authors:** Kangbao Wang, Litao Ma, Chen Yang, Zeyu Bian, Dongdong Zhang, Shuai Cui, Mingliang Wang, Zhe Chen, Xianfeng Li

**Affiliations:** 1 School of Materials Science and Engineering, Shanghai Jiao Tong University, Shanghai 200240, China; kbwang21@sjtu.edu.cn (K.W.); litao.ma@sjtu.edu.cn (L.M.); brucelee75cn@sjtu.edu.cn (X.L.); 2 State Key Laboratory of Metal Matrix Composites, Shanghai Jiao Tong University, Shanghai 200240, China; zhe.chen@sjtu.edu.cn; 3 Innovation Academy for Microsatellites of Chinese Academy of Sciences, Shanghai 201203, China; bianzeyu@microsate.com (Z.B.); zhangdd@microsate.com (D.Z.); cuis@microsate.com (S.C.); 4 School of Physics and Lectronic Information College, Huaibei Normal University, Huaibei 235000, China; 5 Institute of Alumics Materials, Shanghai Jiao Tong University (Anhui), Huaibei 235000, China

**Keywords:** neutron shielding materials, gadolinium (Gd), inorganic nonmetallic materials, polymer materials, metallic materials

## Abstract

With the rising demand for nuclear energy, the storage/transportation of radioactive nuclear by-products are critical safety issues for humans and the environment. These by-products are closely related to various nuclear radiations. In particular, neutron radiation requires specific protection by neutron shielding materials due to its high penetrating ability to cause irradiation damage. Herein, a basic overview of neutron shielding is presented. Since gadolinium (Gd) has the largest thermal neutron capture cross-section among various neutron absorbing elements, it is an ideal neutron absorber for shielding applications. In the last two decades, there have been many newly developed Gd-containing (i.e., inorganic nonmetallic-based, polymer-based, and metallic-based) shielding materials developed to attenuate and absorb the incident neutrons. On this basis, we present a comprehensive review of the design, processing methods, microstructure characteristics, mechanical properties, and neutron shielding performance of these materials in each category. Furthermore, current challenges for the development and application of shielding materials are discussed. Finally, the potential research directions are highlighted in this rapidly developing field.

## 1. Introduction

**Figure 1:**
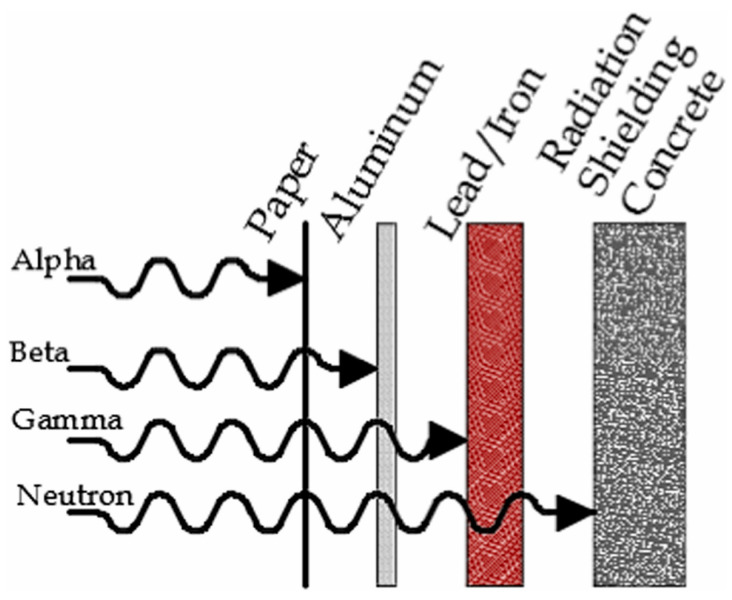
The relative penetrative ability diagram of different radiations [5]. Reproduced with permission from Tyagi et al., Journal of Hazardous Materials; published by Elsevier, 2021.

Over the past few decades, the increasing application of nuclear energy has brought a great deal of convenience to the world. Nuclear energy, as a sustainable and environmentally friendly energy source, plays a critical role in worldwide power generation. It provides around 10% of total global energy output while releasing less greenhouse gas emissions than many other energy sources [1]. Nuclear technology has effectively solved the contradiction between energy demand and environmental pollution. Despite these benefits, the application of nuclear energy is still restricted by a variety of significant challenges. For instance, nuclear power plants simultaneously generate a large amount of highly radioactive by-products (i.e., spent nuclear fuels (SNFs)) in addition to electricity [2]. Herein, SNFs refer to the irradiated nuclear fuel in a nuclear reactor, and they contain various types of radiation (i.e., alpha (α)-rays, beta (β)-rays, gamma (γ)-rays, and neutrons) [3,4,5]. Among these nuclear radiations, neutron radiation should receive priority attention due to its extremely high penetration and irradiation damage ability [4]. Generally, the relative penetrative ability of different radiations is given in Figure 1.

Exposure to neutron radiation can pose serious risks to human health, potentially causing cancer, leukemia, and even genetic mutations [6,7]. Furthermore, the impact of radiation on the natural environment is far-reaching, affecting not only wildlife but also soil and water quality [8]. Given the potential risks of neutron radiation exposure, promoting research and development of neuron shielding materials becomes necessary for protecting humans from the hazards of radiation damage.

**Figure 2:**
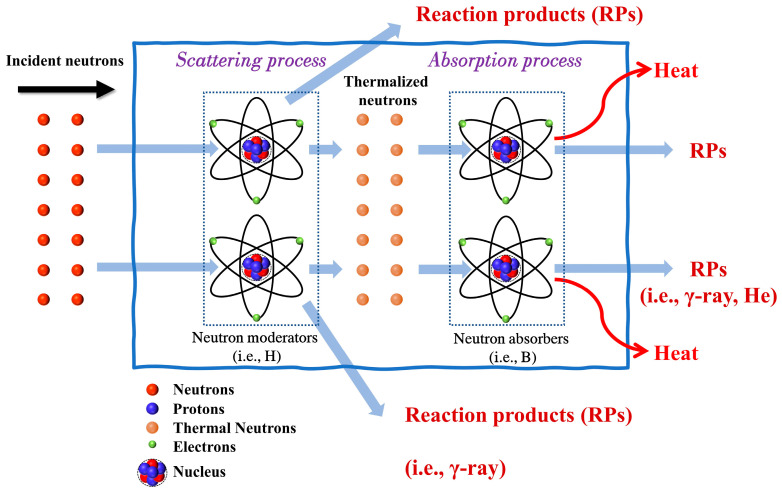
Schematic diagram of the neutron shielding process: scattering and absorption.

Unlike shielding α-, β-, and γ-ray radiation, materials with high atomic numbers and high density are ineffective in neutron shielding. Since neutrons are subatomic particles with no charge and can interact only with the nucleus of target atoms, the likelihood of a specific type of interaction depends on two key factors: the neutron energy and the intrinsic properties of the target nuclide [9]. Neutrons are normally confined within the atomic nucleus. The liberation process necessitates surpassing the binding energy inherent to neutrons, which typically ranges between 7 and 9 MeV for most isotopes. Thus, these free neutrons are initially born with high velocities. According to neutron energy levels, there are typically slow neutrons (energies < 1 MeV), fast neutrons (energies > 1 MeV), and high-energy neutrons (energies > 20 MeV). In particular, thermal neutrons have an energy of 0.0253 eV, equivalent to a velocity of 2200 m/s, and are in thermalized equilibrium with the surrounding atoms at room temperature [10]. Moderation, a vital process, involves the reduction of the initial high speed and kinetic energy of free neutrons through elastic or inelastic scattering. These thermal neutrons are then absorbed by a target nucleus. Neutron absorption is a nuclear process in which an atomic nucleus collides and mixes with one or more neutrons to generate a new nucleus. In brief, the interaction between neutrons and matter is divided into two main parts, namely, moderation (elastic or inelastic scattering) and absorption [11]. It is worth noting that the absorption of fast/high-energy neutrons is nearly impossible due to their high energy and velocity. Comparably, slow neutrons (particularly thermal neutrons) can be easily captured by most neutron absorbers. A schematic diagram of the neutron shielding process is given in Figure 2. In the neutron shielding process, fast neutrons must be moderated/slowed down by elastic or inelastic scattering to become thermal neutrons, which is the process of thermalization or moderation. Subsequently, the thermalized neutrons can be captured by neutron absorbers.

Here, neutron absorption plays an important role in the whole shielding process. The probability that a nucleus will absorb a neutron is defined as the neutron capture cross-section (σ), which has the unit 10^−24^ cm^2^ or “barns” [12]. Neutron shielding materials are often composed of low-atomic-number elements (e.g., H), which have a strong neutron scattering cross-section and may effectively moderate or thermalize the incoming fast neutrons. In addition to this, neutron absorbing elements are generally integrated into neutron shielding materials to absorb the thermalized neutrons. The effectiveness of shielding materials in protecting against neutron radiation is primarily determined by neutron absorbing elements and their concentrations. Normally, a larger neutron capture cross-section corresponds to a higher neutron capture ability. Table 1 summarizes several typical neutron absorbing elements (boron (B), cadmium (Cd), samarium (Sm), and gadolinium (Gd)) with a large neutron capture cross-section.

Among these neutron absorbing elements, Cd is a good thermal neutron absorber, comprising various stable isotopes. ^113^Cd, one of the isotopes of Cd, has a large thermal neutron capture cross-section (σ = 20,600 barns). Previously, Cd was widely utilized in nuclear power reactors due to its favorable processability and low cost. Currently, its application has been constrained due to its high biological toxicity [14]. Additionally, ^149^Sm possesses a higher thermal neutron capture cross-section of 40,000 barns. Florez et al. [15] observed that adding Sm_2_O_3_ to Portland cement substantially boosted thermal neutron attenuation. However, its inherent radioactivity and rapid burn-up render it ineffective in thermal neutron absorption, thus limiting its applications as neutron shielding materials [16].

In contrast, ^10^B is a chemically stable and cost-effective element with outstanding shielding performance as a neutron absorber. It can effectively absorb thermal neutrons through the ^10^B(n, α)^7^Li transmutation reaction [17]. Without the secondary radioactivity caused by neutron absorption, B has gained popularity and is commonly employed in SNF storage [18]. Boron alloy steel [19], hexagonal boron nitride (BN) [20], and boron carbide (B_4_C) [21] are well-known examples of B-containing shielding materials. The addition of B-containing phases can greatly enhance the neutron absorbing performance of the shielding materials, but it comes at the cost of deteriorated plasticity [22]. Moreover, the neutron absorption processes that occur in B-containing phases have the potential to generate helium (He), which may lead to material swelling and cracking [23]. Considering the limitations of conventional boron materials, researchers are looking into potential alternatives to improve the mechanical properties and neutron absorbing ability of shielding materials. Currently, it is still an open question whether to improve the neutron shielding performance of materials with the limited addition of neutron absorbing elements.

One promising strategy is to introduce elements with a larger neutron capture cross-section in the shielding materials, which can effectively reduce the addition of neutron absorbers while simultaneously improving the mechanical properties of the materials. As early as 1935, Dunning et al. [24] discovered that the rare earth element Gd possessed a very high neutron capture cross-section, which was confirmed in subsequent studies [25,26]. For instance, ^157^Gd has the highest neutron capture cross-section of 254,000 barns, which is ~67 times higher than the ^10^B isotope. Another stable isotope, ^155^Gd, also exhibits an extremely high neutron capture cross-section of 62,540 barns. Notably, the total abundance of both isotopes is up to 30%. As a result, Gd has received increased attention in the development of neutron shielding materials. For instance, Burke et al. [27] manufactured gadolinium oxide paint to serve as the neutron attenuation/absorption medium in order to enhance radiation shielding efficiency. The neutron absorption processes of ^155^Gd and ^157^Gd are realized through ^155^Gd(n, γ) and ^157^Gd(n, γ) reactions [28].

**Figure 3:**
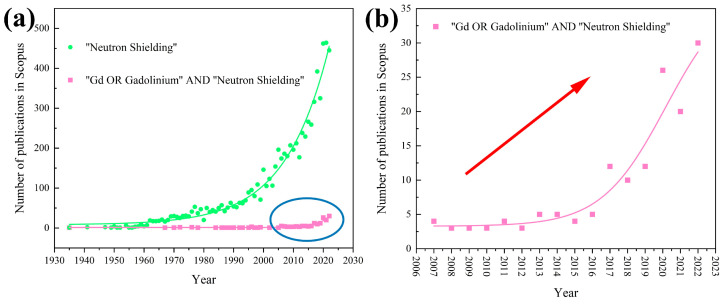
(**a**) The number of publications in neutron shielding and Gd-related neutron shielding for the period spanning 1935–2022, based on the Scopus database. (**b**) The recent trend of Gd-related neutron shielding research ranging from 2007 to 2022 (magnified view in the blue box from Figure 3a).

With the ever-present safety concerns surrounding the advancement of nuclear energy, the field of neutron shielding research has gained increasing interest (Figure 3a). Among a vast number of neutron absorbers, gadolinium (Gd) has an exceptional ability to shield thermal neutrons. As a result, in recent years, there has been a notable upsurge in the publication of novel shielding materials containing Gd, specifically tailored for neutron shielding applications (Figure 3b).

Despite the growing interest in neutron shielding research, systematic reviews focusing specifically on Gd-containing shielding materials remain scarce. Considering the broad prospects of Gd in the field of neutron shielding, we believe that a systematic summary of Gd-containing shielding materials is necessary. Therefore, we present an in-depth review of recent advancements in the design, synthesis, and characterization of Gd-containing shielding materials. This in-depth examination covers three main material categories, including inorganic nonmetallic-based materials (i.e., concrete, glass, and ceramics), polymer-based materials, and metal-based materials (i.e., Fe-based, Al-based, and other metal-based materials). Through this review, we plan to offer a comprehensive diagram of the mechanical and functional properties of the novel Gd-containing neutron shielding materials, their practical challenges, and future prospects in neutron shielding.

## 2. Gd-Containing Inorganic Nonmetallic Neutron Shielding Materials

Inorganic nonmetallic materials (i.e., concrete, glass, and ceramics) are a collective term for materials excluding organic polymers and metals. Comparably, inorganic nonmetallic materials provide considerable economic, chemical stability, and durability benefits, making them highly promising candidates in the field of building and construction (e.g., nuclear reactor buildings). For their applications in nuclear facilities, inorganic nonmetallic materials must possess a certain level of strength and radiation shielding ability. This section focuses on the recent scientific advances in the use of Gd as a neutron absorber in the form of concrete-, glass-, and ceramic-based shielding materials.

### 2.1. Concrete-Based Neutron Shielding Materials

Concrete is a commonly used radiation shielding material mainly against high-energy γ-rays due to its high density, but its shielding ability for thermal neutrons is relatively low [29,30]. Typically, the thickness and weight of the concrete must be quite considerable to enhance radiation shielding performance, which constrains the development of shielding concretes at the same time. Unfortunately, the modification of the water-to-cement ratio of concretes proved unfeasible to improve the shielding performance for incident neutrons [31].

Alternatively, several reports indicated that neutron shielding performance can be improved by incorporating shielding components (e.g., Gd) into the concrete [32]. For instance, using a melt-spinning process, Park et al. [33] prepared the amorphous Fe-B-Mo-Gd ribbons with ~10 wt%Gd, and used them as reinforcements to the concrete matrix. Their findings revealed that the neutron shielding efficiency of the reinforced concrete increased by 86%. Such superior performance can be attributed to the uniform distribution of Gd-containing ribbons within the concrete matrix, effectively absorbing incident neutrons. Furthermore, the reinforced concrete exhibited higher ductility than the original matrix, which was a desirable property for practical applications. In addition, Seshadri et al. [34] performed the transmission measurements of neutrons through Gd-containing concrete, indicating that the dose transmission factor was reduced by ~30% in the 1 wt%Gd/concrete. Nevertheless, with a total shield thickness of 50 cm, the dose rates of Gd-containing concrete were 2.5 times greater than those of ordinary concrete, owing to the excessive generation of γ-rays during the neutron absorption process of Gd. Given the complex nature of harmful radiation, protection against mixed types of radiation, particularly γ-rays and neutrons with strong penetrating properties, has become a key research direction.

Gd_2_O_3_ is a typical oxidized form of the Gd element. As a ceramic reinforced particle, Gd_2_O_3_ is found to be an effective additive as a thermal neutron absorber. For instance, Piotrowski et al. [35] evaluated the neutron shielding properties of ordinary and heavy-weight magnetite concrete modified by epoxy resin and Gd_2_O_3_. Unfortunately, with Gd_2_O_3_ alone, the shielding effectiveness of concrete against fast neutrons was limited. In comparison, a combination of Gd_2_O_3_ and epoxy resin can effectively improve shielding abilities against both fast and thermal neutrons [36]. In their subsequent research, the comprehensive effect of Gd_2_O_3_ addition on Portland cement was discussed in detail [37]. Although Gd_2_O_3_ generated a slight retardation of cement hydration, there was an increased heat intensity released by the acceleration of aluminate activity. Furthermore, Gd-containing concretes not only exhibited enhanced neutron shielding ability, but also showed improved long-term (56 days) compressive and flexural strength, despite a minor drop in strength at the early stage (3 days). Long-term (56 days) compressive and flexural strengths were 4% and 15% higher than the reference specimens, respectively.

To summarize, the integration of Gd into concrete has shown significant potential in the area of neutron shielding. Considering the limited shielding ability of Gd against fast neutrons, polymer materials with a high H content are often used together with Gd-containing shielding concretes to fill this gap. This approach provides a viable future option for improving the neutron shielding performance of Gd-containing concretes.

### 2.2. Glass-Based Neutron Shielding Materials

Glass is a non-crystalline and amorphous solid that has many advantages, such as high optical transparency, high chemical stability, and low cost. Glasses with high transparency outperform other materials for doors, windows, and screens in scintillators, nuclear power plants, and medical diagnostic or treatment rooms [38,39]. Gd is a paramagnetic element with a high atomic number (64) and density (7.895 g/cm^3^). From an overall standpoint, Gd is a good choice for the application of radiation shielding glasses. In recent studies [40,41,42,43,44,45,46,47,48,49,50,51], Gd-containing shielding glasses have mainly focused on γ-ray radiation shielding ability, which should be discussed in the following section. Considering that Gd-containing shielding materials tend to release highly hazardous secondary γ-rays while absorbing neutrons, this type of work should be helpful to improve the comprehensive shielding performance of Gd-containing neutron shielding glasses.

Kaewjang et al. [40] reported Gd_2_O_3_/B_2_O_3_-SiO_2_-CaO glasses for γ-ray radiation shielding. All Gd_2_O_3_/glass samples showed a lower half-value layer (HVL, the thickness of a material that can attenuate 50% of the initial intensity of radiation) than ordinary concrete, X-ray shielding windows, and commercial windows. Here, low HVL represents good γ-ray shielding performance. Furthermore, the increased Gd_2_O_3_ content led to a linear increase in the glass density due to its high molecular weight [41,42]. Glasses with a higher density are useful in radiation shielding against γ-rays [43]. When it comes to neutron shielding performance, *x*Gd_2_O_3_/B_2_O_3_-10SiO_2_-10CaO (*x* = 15, 20, 25, 30, and 35 mol%) fabricated by melt quenching exhibited different neutron shielding performances depending on the neutron energy [42]. The 15 mol%Gd_2_O_3_/glass is a superior shielding material for neutron energy of 1 eV, and the 35 mol%Gd_2_O_3_/glass becomes the optimum choice at >2 MeV neutron energy.

In addition, Al-Buriahi et al. [44] surveyed the impact of Gd_2_O_3_ addition on γ-rays and neutron shielding capacities of TeO_2_-ZnO-Nb_2_O_5_ glasses and found that the fast neutron removal cross sections (∑_R_) of glasses were improved with the Gd_2_O_3_ addition, indicating their good fast neutron attenuation ability. They attributed this improvement to the increased density due to the replacement of Nb_2_O_5_ by Gd_2_O_3_. Nevertheless, the thermal neutron shielding properties of Gd_2_O_3_/TeO_2_-ZnO-Nb_2_O_5_ glasses were not studied here. Similarly, Al-Hadeethi et al. [45] observed that ∑_R_ value was raised with the Gd_2_O_3_ addition to TeO_2_-ZnO-Nb_2_O_5_ glasses, but this improvement was rather limited. They held the view that the improvement of fast neutron attenuation was derived from the density change of shielding glasses.

**Figure 4:**
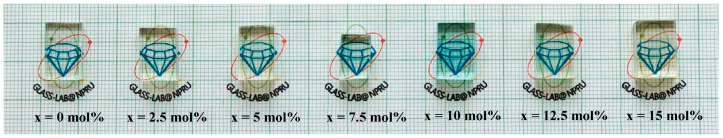
Photographs of several gadolinium sodium borosilicate glasses with Gd_2_O_3_ content varying from 0 mol% to 15 mol% [50]. Reproduced with permission from Intachai et al., Radiation Physics and Chemistry; published by Elsevier, 2022.

Moreover, Ibrahim et al. [46] fabricated ZnO-Bi_2_O_3_-B_2_O_3_ glasses doped with rare earth oxides (e.g., La_2_O_3_, CeO_2_, Nd_2_O_3,_ and Gd_2_O_3_) by a melt quenching process. Among these glasses, Gd_2_O_3_/glass had the highest oxygen packing density, revealing that the structure of the Gd_2_O_3_/glass composite was tightly packed. Besides, Gd_2_O_3_/glass has the characteristics of higher density, lower molar volume, and higher γ-ray radiation shielding efficiency. The fast neutron removal cross-section (∑_R_) followed the order ∑_R_ (Gd_2_O_3_/glass) > ∑_R_ (Nd_2_O_3_/glass) > ∑_R_ (Ce_2_O_3_/glass) > ∑_R_ (La_2_O_3_/glass) > ∑_R_ (B_2_O_3_/glass). The best fast neutron attenuation ability was achieved for Gd_2_O_3_/glass with ∑_R_ = 0.1 cm^−1^, which was higher than that of ordinary concretes, hematite serpentine concretes, and ileminite-limonite concretes. Their results revealed that Gd_2_O_3_ had distinct advantages in the field of radiation shielding over other rare earth oxides (La_2_O_3_/CeO_2_/Nd_2_O_3_). According to Lakshminarayana’s report [47], the 37.5 mol%Gd_2_O_3_/SiO_2_-B_2_O_3_ glass showed a large total neutron cross-section (σ_T_) of 646.171 cm^−1^, indicating that Gd_2_O_3_ plays an important role as a thermal neutron shielding component. Overall, the Gd_2_O_3_ addition dramatically improved the thermal neutron shielding performance of glasses, but its influence on attenuating fast neutrons required further investigations [44,45,48]. Critically, the addition of Gd_2_O_3_ has a minimal impact on the light transparency of radiation-shielding glasses, as evidenced by previous studies [40,49,50]. For instance, gadolinium sodium borosilicate glasses demonstrated a negligible reduction in light transmission with increasing Gd_2_O_3_ content [50], as visually observed in Figure 4. These findings highlight the significant potential of Gd_2_O_3_-containing glasses in the field of radiation shielding, as they retain excellent light transparency properties.

Apart from Gd_2_O_3_-doped shielding glasses, gadolinium oxyfluoride (Gd_4_O_3_F_6_)-doped glass is also an applicable radiation shielding glass system. However, both radiation attenuation parameters and HVL values of Gd_4_O_3_F_6_/glass are inferior to those of Gd_2_O_3_/glass [51], leading to its limited use in radiation shielding. Nevertheless, both shielding glass systems exhibited good γ-ray shielding properties and high light transmittance. However, there is relatively little research focusing on the neutron shielding properties of Gd-containing glasses. For applications, Gd can effectively absorb thermal neutrons, but it has a limited contribution to fast neutron attenuation. In addition, it is known that the γ-ray shielding performance of Gd-containing glasses improves with increased Gd content. Nevertheless, little research has been undertaken to comprehensively analyze the influence of excessive Gd on glass systems.

### 2.3. Ceramic-Based Neutron Shielding Materials

Ceramics are a group of hard, heat- and corrosion-resistant materials created under heat and/or pressure conditions, consisting of at least two elements (one of which must be non-metallic) [52]. Considering their applications as structural components for fusion reactors, cladding for gas-cooled fission reactors, and immobilized forms for radioactive waste [53], it is imperative to design ceramics with good neutron and radiation shielding performance.

Currently, there are several related studies on Gd-containing radiation shielding ceramics. For example, when Ge et al. [54] prepared Gd_2_O_3_/Al_2_O_3_ ceramics by pressure-less sintering, they found that the γ-ray radiation shielding property of materials improved with the increased Gd_2_O_3_ content. However, both mechanical properties and thermal conductivities decreased significantly when the Gd_2_O_3_ content exceeded 15 wt%. Besides, excessive new phases (i.e., Gd_2_Si_2_O_7_, Al_2_Gd, and Al_5_Gd_3_O_12_) formed under this condition, hindering the densification of ceramics. The conflicts between shielding performance and mechanical properties should require more attention in further research. Hernandez et al. [55] fabricated Gd_2_O_3_/bauxite ceramics by sintering procedures and found that their macroscopic neutron capture sections (∑) were linearly correlated with the Gd_2_O_3_ incorporation. The 10 wt%Gd_2_O_3_/bauxite ceramics exhibited the highest neutron capture section of 1.5 × 10^5^ barns, and their corresponding mechanical properties were also excellent compared with other ceramics.

Perovskite metal oxides (e.g., ABO_3_ structures) have attracted significant attention recently due to their potential use in various applications (e.g., catalytic responses, piezoelectricity, and photoelectricity sensors) [56]. Gadolinium borate (GdBO_3_) is an important binary compound in the Gd_2_O_3_-B_2_O_3_ phase diagram, and also a proper candidate for neutron shielding. Dong et al. [57] synthesized the GdBO_3_ ceramics by pressure-less sintering from 950 to 1100 °C using Gd_2_O_3_ and boric acid. The results indicated that GdBO_3_ ceramics possessed high thermal stability, low thermal conductivity, and good neutron shielding performance. Additionally, their neutron shielding ability was highly correlated with Gd_2_O_3_ content, sintering temperature, and sample thickness. Moreover, the B content can also contribute to the neutron shielding performance of GdBO_3_ due to its neutron absorbing ability. Li et al. [58] evaluated the structural stability and neutron-shielding capacity of GdBO_3_/Al_18_B_4_O_33_ ceramics fabricated by a pressureless sintering process. In comparison to Gd-free ceramics, the 6 wt%Gd_2_O_3_ addition brought a striking decrease in neutron transmission rate (i.e., from 65% to 26%) to the shielding composite at a fixed sample thickness of 0.5 cm. This dramatic downward trend can be attributed to the Gd_2_O_3_ incorporation with an optimal addition (6 wt%) to improve the structural stability and mechanical properties of ceramics. Nevertheless, further Gd_2_O_3_ addition should lead to excessive GdBO_3_ formation, resulting in intergranular micro-cracks. This was detrimental to the comprehensive properties of neutron shielding ceramics.

High-entropy ceramics are solid solutions of five or more cation or anion sublattices, with a high configuration entropy. Recently, these systems have become increasingly popular due to their diversity in compositional design, which can be tailored to meet specific needs in different application scenarios [59]. For instance, Zhang et al. [60] designed and synthesized a novel monoclinic-type high-entropy ceramic (La_0.2_Ce_0.2_Gd_0.2_Er_0.2_Tm_0.2_)_2_ (WO_4_)_3_ powder by high-temperature sintering and ball milling processes. Due to the improved phase stability offered by the high entropy effect, the powder was less likely to fail when exposed to radiation. Gd acted as an effective neutron absorber in this high-entropy system. Then, they fabricated the shielding ceramic by dispersing (La_0.2_Ce_0.2_Gd_0.2_Er_0.2_Tm_0.2_)_2_(WO_4_)_3_ powder in epoxy resin, and the ceramic composite showed an acceptable tensile strength (=19.25 MPa), relatively lower thermal conductivity (<0.3 W·m^−1^·K^−1^), and density (<1.5 g·cm^−3^). Importantly, such a composite system with the highest content of high-entropy ceramic powders can absorb most thermal neutrons.

**Figure 5:**
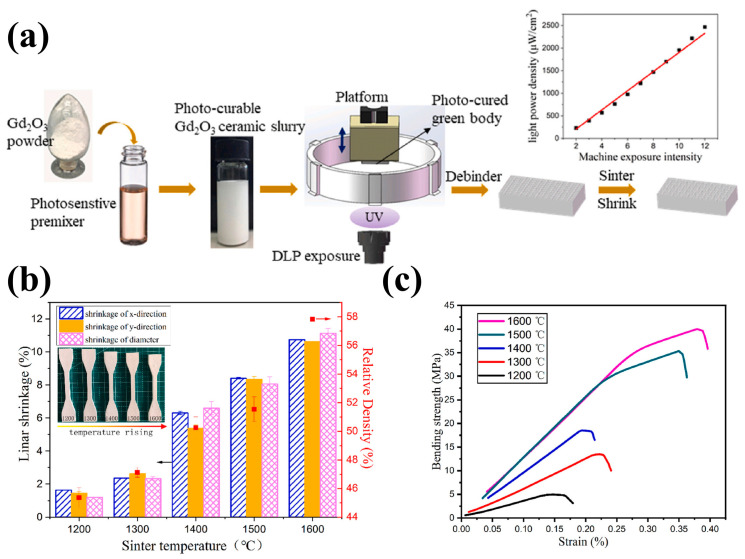
Preparation, characteristics, and mechanical properties of as-printed Gd_2_O_3_ ceramics [61]. (**a**) Schematic of the vat photopolymerization 3D printing process for Gd_2_O_3_ ceramics. (**b**) Diagrams illustrating relative densities and linear shrinkage to sinter temperature. (**c**) The bending strength curves of as-printed ceramics as a function of strain with the increasing sintering temperature. Reproduced with permission from Wang et al., Ceramics International; published by Elsevier, 2022.

For fabricating ceramic shielding composites with unique designs and customized requirements, the additive manufacturing technique offers more freedom compared with the traditional milling, curving, and injection molding processes. Based on these considerations, Wang et al. [61] manufactured the Gd_2_O_3_ ceramics by vat photopolymerization 3D printing (Figure 5a). They found that both linear shrinkage (Figure 5b) and mechanical properties (Figure 5c) of as-printed ceramics increased with higher sintering temperatures. With a sintering temperature of 1600 °C, the as-printed ceramics achieved a density of 58%, a bending stress of 40 MPa, and a flexural elastic modulus of 20.2 GPa. Although there are still some problems with the formability of as-printed ceramics, this study provides a good strategy for the design and preparation of ceramic-based shielding materials.

## 3. Gd-Containing Polymeric Neutron Shielding Materials

**Figure 6:**
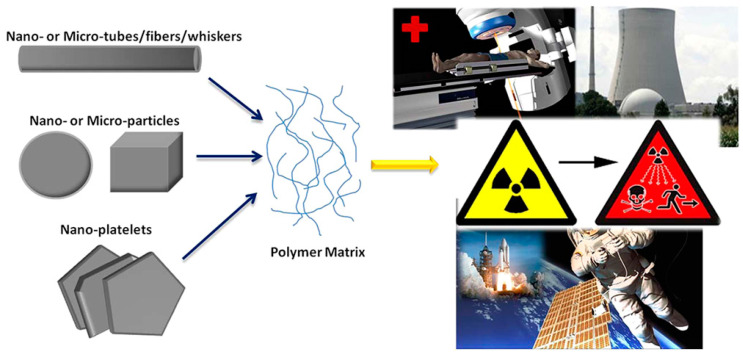
Filler-reinforced polymer composite materials for effective radiation protection [66]. Reproduced with permission from Nambiar et al., Applied Materials; Copyright (2019) American Chemical Society.

A polymer is a substance or material consisting of macromolecules, which are made up of many repeating subunits. The H element is an important component for neutron shielding since it can easily thermalize the incident neutrons with high energy and velocity through the elastic scattering process [62]. Polymer matrix composites (PMCs) are high-H-content materials. They can effectively attenuate fast neutrons, which makes them an attractive candidate for neutron shielding [63]. Generally, PMCs have remarkable advantages, such as being flexible, cost-effective, lightweight, and corrosion-resistant [64]. Most importantly, the neutron shielding performance of PMCs can be improved by introducing neutron absorbers (e.g., B or Gd) as shielding fillers in the polymer matrix [65]. Therefore, they have great potential for several radiation shielding applications (e.g., wearable protective devices for the nuclear or aerospace industries) (Figure 6) [66]. For instance, Yastrebinskii et al. [67] successfully designed a neutron-protective PMC cover with a high concentration of Gd filler for transporting packing containers of SNFs. This section focuses on Gd-containing PMCs and their potential in neutron shielding applications, as well as the effect of Gd particle size on the neutron shielding performance of these PMCs. In addition, some notable research advances will be mentioned in this area.

### 3.1. Traditional Polymer-Based Shielding Neutron Materials

In recent years, various polymers have been developed as neutron shielding materials, among which epoxy resin (ER), polyvinyl alcohol (PVA), and polyethylene (PE) are notable examples [64]. Normally, polymer materials can attenuate fast neutrons through the scattering process, achieving the desired neutron shielding efficiency at the required thickness of shielding materials. However, the thermal neutron shielding properties of polymer materials are relatively limited. Therefore, the incorporation of Gd, which has the highest thermal neutron capture cross-section, is a promising approach to improving the shielding performance of polymer materials.

PVA, ([CH_2_CH(OH)]_n_), which is a H- and O-rich polymer macromolecule with self-healing ability, can effectively attenuate fast neutrons [68]. Based on this point, several PVA-based shielding composites were developed. To improve their thermal neutron shielding performance, Tiamduangtawan et al. [69] introduced modifications to PVA hydrogels with either Sm_2_O_3_ or Gd_2_O_3_ fillers. Their findings revealed that both Gd_2_O_3_/PVA and Sm_2_O_3_/PVA hydrogels exhibited substantially enhanced thermal neutron capture abilities. Furthermore, both hydrogels displayed autonomous self-healing capacity on damaged/fractured surfaces. Additionally, these hydrogels showed increased tensile modulus and strength. Nevertheless, a small decrease in elongation occurred with the addition of Gd_2_O_3_ and Sm_2_O_3_ fillers. In terms of thermal neutron shielding effectiveness and tensile properties, Gd_2_O_3_/PVA hydrogels outperformed Sm_2_O_3_/PVA hydrogels under the same filler concentration and thickness of shielding composites.

Additionally, Wang et al. [70] synthesized poly(MMA-co-Gd(MAA)_3_) through the self-polymerization process of gadolinium methacrylate (Gd(MAA)_3_). The results indicated that poly(MMA-co-Gd(MAA)_3_) outperformed poly(methyl methacrylate) (PMMA) in thermal neutron shielding. To achieve a thermal neutron shielding efficiency of 90%, ~70 mm thick PMMA was required. Comparably, to achieve such a similar effect, the poly(MMA-co-Gd(MAA)_3_ only needed the thickness at ~14 mm (0.38 wt%Gd), ~5 mm (1.91 wt%Gd), and ~2.4 mm (3.81 wt%Gd). Moreover, Gd(MAA)_3_ can also be applied to polyacrylonitrile (PAN) as a thermal neutron absorber [71].

In a practical nuclear radiation environment, high-energy neutron and γ-ray radiation normally occur simultaneously. Besides, Gd also releases secondary γ-ray radiation when it absorbs neutrons. Thus, the design of Gd-containing neutron shielding must consider γ-ray radiation protection as well. Some investigations of Gd-containing neutron shielding materials often neglect this aspect, which needs more attention in further studies. Recently, Wang et al. [72] synthesized Gd_2_O_3_/polystyrene-block-poly(ethylene-ran-butylene)-block-polystyrene (SEBS) composites for thermal neutron and γ-ray radiation shielding applications. In their research, the Gd_2_O_3_ particles were homogeneously and tightly connected to the matrix via a melting and blending process. Additionally, the Gd_2_O_3_/SEBS composites exhibited high flexibility. By comparing the neutron transmittance rates of Gd_2_O_3_/SEBS and B_4_C/SEBS composites, they found that Gd_2_O_3_ was a superior neutron absorber in the 0.025–0.15 eV range over B_4_C. Experimental results revealed a positive correlation between neutron shielding performance and Gd_2_O_3_ addition, as evidenced by a decrease in neutron transmittance from 59.1% to 35.3% as Gd_2_O_3_ content increased from 3.9 wt% to 54 wt%. In their study, they found that the Gd_2_O_3_/SEBS composite had the best shielding efficiency for γ-rays at 59 keV. Specifically, γ-ray transmittance reduced with increasing Gd_2_O_3_ concentration, and the transmittance may be lowered from 90.08% to 28.42% with the introduction of Gd_2_O_3_ for 59 keV γ-rays.

The idea of self-healing is a very promising strategy for increasing the lifespan of some polymers by enabling them to repair physical damage on their own [73]. For instance, both Gd_2_O_3_/PVA and Sm_2_O_3_/PVA hydrogels designed by Tiamduangtawan et al. [69] exhibited autonomously self-healing properties at fractured surfaces. These hydrogels were able to recover a portion of their mechanical strengths over time, but the recovery efficiency was reduced with increased concentrations of Sm_2_O_3_ or Gd_2_O_3_. For self-healing shielding materials, Poltabtim et al. [74] proposed a Gd_2_O_3_-reinforced natural rubber (NR) composite. Such a Gd_2_O_3_/NR composite exhibited remarkable self-healing capability at fractured surfaces due to the incorporation of a reversible ionic supramolecular network. The addition of Gd_2_O_3_ to NR led to a substantial enhancement in neutron shielding performance. Even a minor addition of 25 parts per hundred rubber (phr) of Gd_2_O_3_ to pristine NR resulted in a drastic reduction in both thermal neutron attenuation rate (I/I_0_) and half-value layer values (HVL) over the NR matrix. Specifically, I/I_0_ and HVL were reduced from 97% and 84 mm in pure NR to a mere 55% and 2.3 mm in the 25 phr Gd_2_O_3_/NR composite (both I/I_0_ and HVL values were compared using 2 mm thick samples). However, the Gd_2_O_3_/NR composite showed decreased mean tensile strength and elongation with increasing Gd_2_O_3_ addition, but increased hardness. In summary, the available results demonstrate that the self-healing shielding materials that have been investigated thus far must have a better recovery efficiency. Since shielding materials are subjected to a variety of damages during service, the development of neutron shielding PMCs with good self-healing capability is of great importance.

### 3.2. Novel Polymer-Based Shielding Neutron Materials

**Figure 7:**
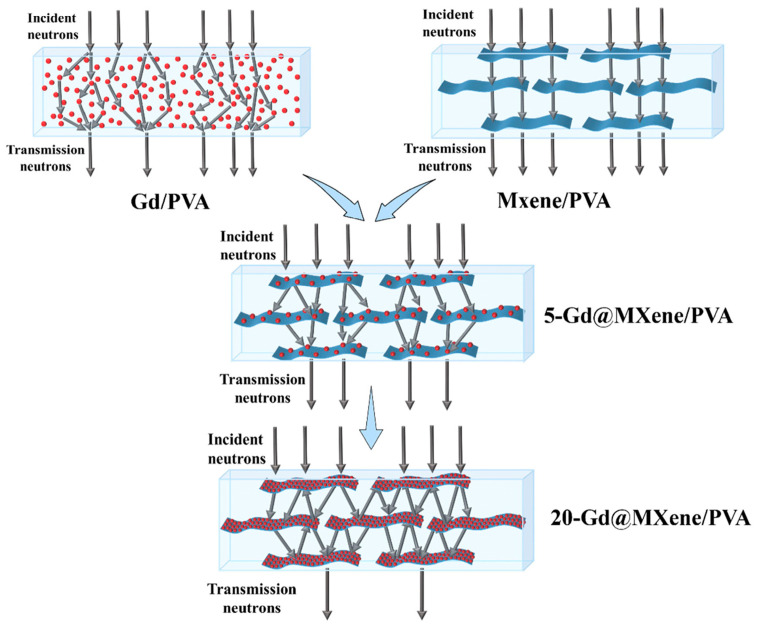
Diagram of the neutron shielding process of four modified PVA-based composites [76]: Gd/PVA, MXene/PVA, 5-Gd@MXene/PVA, and 20-Gd@MXene/PVA. Reproduced with permission from Zhu et al., Nanoscale; published by the Royal Society of Chemistry, 2022.

MXenes are a new family of 2D transition metal carbides, carbonitrides, and nitrides. Due to their favorable 2D layered structure, highly tunable composition, and surface functional groups, MXenes have drawn significant interest in a variety of applications, including sensors, energy storage, and shielding from electromagnetic interference [75]. Inspired by fish scales, Zhu et al. [76] utilized the spin coating technique to produce Gd@MXene/PVA films, as formed by PVA and Gd@MXene nanoflakes (Figure 7). The Gd@MXene nanoflakes were synthesized through a hydrothermal reaction and consisted of 2D Ti_3_C_2_T_x_-MXene nanoflakes and 0D Gd nanoparticles. Their design aligned the random distribution of Gd nanoparticles into an oriented 2D pattern, creating fish scale-like barrier walls. These walls effectively scattered neutrons multiple times between the nanoflakes, enhancing the neutron shielding efficiency of Gd nanoparticles. In Figure 7, the Gd@MXene/PVA composite showed synergistic effects with improved thermalization and absorption efficiency towards incident neutrons over both Gd/PVA and MXene/PVA composites. Since MXene has limited neutron shielding ability, neutrons can directly permeate the MXene/PVA layer. In comparison, the incorporation of Gd@MXene with the oriented 2D fish scale-like barrier walls facilitated multiple scatterings of slow neutrons. Moreover, the Gd@MXene/PVA composites showed improved neutron shielding properties with increased Gd@MXene contents. The thermal neutron attenuation rate of 20 wt%Gd@MXene/PVA was ~10.44%, which is 3.36 times higher than pure PVA.

Metal–organic frameworks (MOFs) are made by linking inorganic and organic units with strong bonds to create open crystalline frameworks with high porosity [77]. Hu et al. [78] prepared the Gd-MOF-reinforced epoxy resin (EP) composite using a solution casting approach. The hardness, stiffness, and thermal neutron capture ability of the composite increased remarkably with the addition of Gd-MOF fillers. However, the composite showed a slight reduction in tensile strength, potentially attributed to the weak interfacial adhesion between the Gd-MOF fillers and the matrix. Moreover, the incorporation of Gd-MOF fillers improved the thermal stability of the Gd-MOF/EP composite. In particular, the 10 wt%Gd-MOF/epoxy composite showed an acceptable tensile strength of 66.6 MPa and good thermal neutron shielding performance. Similarly, a recent study revealed that Gd-MOF fillers have a high potential for use as polyimide (PI) matrix reinforcements in improving neutron shielding performance [79]. The fast neutron penetration rate of the 3 wt% Gd-MOF/PI composite was 9.6% when the specimen thickness was 0.2 cm, while the thermal neutron penetration rate was only 0.9%. Compared with the original PI material, the introduction of Gd-MOF greatly improved the neutron shielding performance of the polymer matrix. In contrast to pure PI, the Gd-MOF/PI composite had a higher glass transition temperature (Tg), making it a suitable choice for engineering applications. Meanwhile, the Gd-MOF/PI composite exhibited improved thermal stability, which in turn increased the service temperature of the polymer shielding material. Generally, these examples provide valuable insights for designing high-performance neutron shielding materials based on polymers (which exhibit better fast neutron attenuation capability) and Gd fillers (which have effective thermal neutron shielding properties).

### 3.3. Improving Neutron Shielding and Mechanical Properties of PMCs with Gd Nanofillers

The advent of nanotechnology has greatly facilitated the application of nanomaterials as highly effective fillers/reinforcements, resulting in noteworthy enhancements in the mechanical properties and radiation shielding efficiency of composites [67,80]. There are considerable research efforts devoted to the applications of Gd-based nanofillers in PMCs for radiation shielding purposes [63,76]. In this section, the influence of Gd particle size on both radiation shielding and the mechanical properties of PMCs is discussed.

For instance, Li et al. [81] investigated the impact of particle size on the radiation shielding performance of epoxy resin (ER) matrix composites infused with dispersed micro- and nano-Gd_2_O_3_ fillers. The results indicated that nano-Gd_2_O_3_/ER composites had a superior ability to shield X-/γ-rays than their micro-Gd_2_O_3_/ER counterparts. This can be attributed to the fact that the nanoparticles have a smaller size and uniform distribution, increasing the likelihood of interaction between X-/γ-rays and particles. Interestingly, the size effect is strongest at low particle concentrations and declines as particle concentration increases. Prabhu et al. [67] also claimed that the enhancement of radiation shielding efficiency of composites was predominant with Gd nanofillers with decreased size at very low loadings. Although the shielding composites were not tested for neutron absorption in the above two studies [67,81], their findings have offered a new perspective on the role of particle size in material design.

In İrim’s work [82], the high-density polyethylene (HDPE) was reinforced by hexagonal boron nitride (h-BN) and Gd_2_O_3_ nanoparticles using the melt-compounding technique. The as-fabricated h-BN@Gd_2_O_3_/HDPE nanocomposites demonstrated impressive advancements in both neutron and γ-ray radiation shielding performance, leading to enhancements of 200–280% and 14–52%, respectively. Notably, Gd_2_O_3_ served the dual purpose of shielding against both neutron and γ-ray radiation. Simultaneously, the mechanical properties (i.e., tensile strength and modulus) were enhanced by the addition of nanoparticles. Likewise, Baykara et al. [83] incorporated Gd components into PMCs by creating polyimide nanocomposites doped with h-BN and Gd_2_O_3_ nanofillers. The shielding composites exhibited improved mechanical properties and radiation shielding performance, highlighting the potential of Gd nanofillers as versatile reinforcements for the design and development of advanced shielding materials.

The integration of Gd nanofillers in PMCs has brought remarkable improvements in both radiation shielding performance and mechanical properties. Nevertheless, recent studies revealed that excessive Gd content may have unfavorable impacts, i.e., a linear decrease in thermal stabilities of Gd_x_Fe_2_O_3_/PVA composites [84]. Likewise, He et al. [85] reported that the addition of nano-Gd_2_O_3_ is unfavorable to the thermal stability of the salicylic acid polyphenylene sulfide (SAPPS) composites. Additionally, the mechanical properties of nano-Gd_2_O_3_/SAPPS composites were adversely affected by the excessive nanofillers. Given the high temperatures encountered in operation, thermal stability is a key consideration when selecting radiation shielding materials. To improve thermal stability, Huo et al. [86] introduced surface-modified micro- and nano-Gd_2_O_3_ fillers to reinforce HDPE composites. They discovered that the modified fillers enhanced the thermal stability of composites by improving interfacial compatibility, which restricted the movement of HDPE chains. Furthermore, nano-Gd_2_O_3_ had a larger interaction probability with radiative particles (neutrons and γ-rays) due to its high dispersibility in the matrix (HDPE) than micro-Gd_2_O_3_. In addition, the uniform dispersion of nano-Gd_2_O_3_ filler in the matrix reduced the stress concentration effect and increased the tensile strength. Thus, the modified nano-Gd_2_O_3_/HDPE composites showed superior neutron and γ-ray radiation shielding performance and mechanical properties over the modified micro-Gd_2_O_3_/HDPE composites. Both particle size and surface modification improved the radiation shielding performance of composites. To summarize, the size of particles is a crucial factor in enhancing the neutron shielding properties of PMCs. The significant enhancements can be attributed to the highly synergistic interaction between the polymer matrix and nanoparticles. This interaction results in a reinforcing effect that surpasses that of most conventional microparticles. However, the improvement of nanoparticle homogeneity in PMCs remains a topic of ongoing research. Overall, the utilization of Gd nanoparticles as fillers in PMCs offers novel prospects for the design and processing of high-performance shielding materials with targeted properties.

In conclusion, the use of Gd offers significant potential for the advancement of polymer-based neutron shielding materials, and the reported findings are presented in Table 2. This should serve as a reference for the design and research of Gd-containing polymer neutron shielding materials.

## 4. Gd-Containing Metallic Neutron Shielding Materials

With the steady growth of nuclear energy, the development of neutron shielding structural materials for the storage and transportation of SNFs or radioactive wastes is a pressing issue in nuclear engineering and technology nowadays [87]. Practically, shielding materials have to operate under high-energy radiation exposure and multiple corrosive media in either dry or wet circumstances. Thus, ensuring the long-term stability of neutron shielding materials should necessitate not just adequate radiation shielding ability, but also good mechanical properties and corrosion resistance. In this regard, metallic materials offer an obvious advantage over inorganic nonmetallic materials and polymer materials. For example, polymer-based shielding materials cannot withstand higher temperatures (e.g., 200 °C) due to their high H content [88]. In contrast, metallic materials are widely used as structural materials in the storage and transportation of SNFs given their high thermal stability, mechanical strengths, and corrosion resistance [89].

Depending on their compositions, Gd-containing metallic materials can be classified into three categories: Fe-based alloys/composites, Al-based alloys/composites, and other metal-based alloys/composites. In metallic neutron shielding materials, Gd can also be introduced to the matrix in the metallic form as Gd-containing phases in addition to the common Gd_2_O_3_ ceramic reinforcements. Here, we will present the latest developments in metallic neutron shielding materials in each category.

### 4.1. Fe-Based Alloys/Composites

Fe-based alloys/composites, primarily utilizing stainless steel as the matrix, have been developed as neutron shielding materials. Stainless steel, known for its excellent corrosion resistance and mechanical properties, is a major structural material in nuclear reactor systems [90]. Incorporating neutron absorbers into stainless steel has been identified as a viable strategy for the development of Fe-based neutron shielding materials. Borated austenitic stainless steel, for example, has 0.2–2.25 wt%B and offers strong mechanical properties and neutron shielding performance for SNF storage applications [91]. However, due to the poor solubility of the B element in stainless steel, fabricating borated austenitic stainless steel with more than 2.25 wt%B is relatively difficult [92]. Furthermore, the B element would emit He bubbles after absorbing neutrons, which adversely affects its actual application [23].

The Gd-containing steel was first proposed by Kato [93], where Gd was utilized as a neutron shielding phase without the formation of He bubbles during the neutron absorption process. In addition, Gd has a far larger neutron capture cross-section than B. Therefore, the Gd-containing steel can effectively solve the trade-off between alloying contents and neutron shielding performance, allowing for higher neutron shielding performance with less alloying addition. Among available stainless steels, the duplex stainless steels (DSS), comprising both ferrite and austenite phases, exhibit a combination of superior strength and corrosion resistance. For this reason, Choi et al. [94] developed a 1 wt%Gd/DSS produced by melting, casting, hot-rolling, and solution treatment processes. The as-cast steel contained typical duplex phases (i.e., 31% ferrite and 69% austenite), and Gd-rich precipitates were found inside grains and at grain boundaries. After hot rolling to 6 mm thickness, the Gd/DSS exhibited good formability without cracks and showed improved strength and superior stress corrosion resistance. Later, Choi et al. [95] fabricated a 0.087 wt%Gd/DSS shielding material by casting, hot rolling, solution treatment, and cold rolling techniques. The ultimate tensile strength and elongation of the cold-rolled 0.087 wt%Gd/DSS were 694 MPa and 37.1%, which can be compared to the 1 wt%Gd/DSS with a higher content of Gd. A similar study by Baik et al. [96] indicated that the majority of Gd was detected as sub-micron precipitates of GdCrO_3_ in the 0.02 wt%Gd/DSS. When exposed to a neutron beam with a wavelength of 0.48 nm, the neutron absorption coefficient of a 2 mm thick 0.02% wt%Gd/DSS sheet was approximately three times greater than that of pure DSS.

The austenitic stainless steels (e.g., 304 and 316(L)), where austenite is the primary crystalline structure, are another prevalent type of stainless steel. Such stainless steels have higher Ni and Cr contents, offering exceptional formability, weldability, mechanical strength, and corrosion resistance [97]. Due to the low solubility of Gd in both ferrite and austenite phases, the Gd element is mostly formed in the intermetallic phase [98]. Robino et al. [99] designed a (0.1–10 wt%) Gd/316L steel for SNF storage, with the majority of Gd components forming an intermetallic phase of (Fe, Ni, Cr)_3_Gd. This intermetallic phase was identified to contain ~28 wt%Ni and ~3 wt%Cr, resulting in Ni depletion and Cr enrichment in the steel matrix, which in turn influenced the matrix stability. With increased Gd concentration, the formation of the (Fe, Ni, Cr)_3_Gd phase and the resulting shift in matrix structure from austenite to ferrite led to a rise in the hardness of the Gd/316L steel. However, DuPont et al. [100] discovered that the presence of this intermetallic phase in stainless steels posed challenges for both weldability and hot rolling process. Wang et al. [101] found that the Gd-rich phases with an average size of ≤3 μm were uniformly dispersed at ≤1.5 wt%Gd/316L steel after hot rolling. As Gd concentration increased to 2 wt%, some Gd-rich phases coarsened to ~6 μm and agglomerated, which had a detrimental effect on its corrosion resistance. However, there have been few investigations conducted to thoroughly investigate the structure and stability of Gd-rich phases to date.

**Figure 8:**
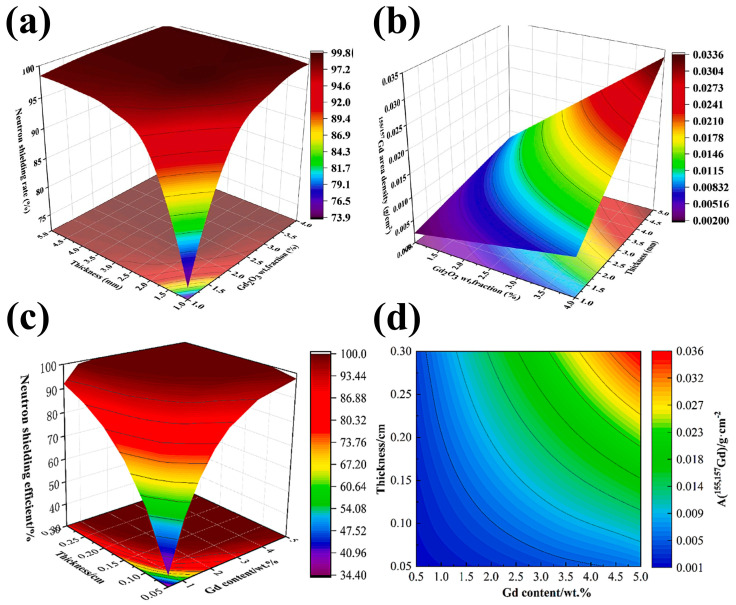
Neutron shielding performance prediction model: MCNP simulation of Gd_2_O_3_/316L composite) [104]. (**a**) 3D plots of MCNP results. (**b**) The 2D contour map of ^155/157^Gd areal density. Reproduced with permission from Wan et al., Vacuum; published by Elsevier, 2020.; MCNP simulation of Gd/316L steel [106]. (**c**) The 3D relationship between thermal neutron shielding rate, Gd content, and material thickness. (**d**) 2D contour maps of A(^155,157^Gd). Reproduced with permission from Qi et al., Materials Today Communications; published by Elsevier, 2023.

The low solubility of Gd, similar to that of B, restricted its applications in Fe-based neutron shielding materials. However, non-equilibrium fabrication processes (e.g., mechanical alloying) can effectively deal with this issue [102]. Yang et al. [103] prepared the 7.87 wt%Gd/316L steel by mechanically alloying Gd_2_O_3_ and 316L powders, followed by hot isostatic pressing. Herein, the nanosized Gd-Si-O particles (~12.5 nm) were evenly distributed in the alloy through a dissolution and re-precipitation mechanism. The 7.87 wt%Gd/316L steel had much better thermal neutron shielding performance than 316L steel. Results showed that the 7.87 wt%Gd/316L alloy with a thickness of 0.2 mm exhibited the equivalent thermal neutron attenuation capability of a much thicker (33 mm) 316L alloy, achieving a 90% reduction in thermal neutron transmission. Their research provided a promising route for fabricating neutron shielding alloys enriched with a high Gd content through the process of mechanical alloying. Although the Gd addition can improve the neutron shielding performance of stainless steels, the relationships between the Gd concentrations, the thickness of shielding materials, and shielding efficiency remain unclear. Wan et al. [104] fabricated Gd_2_O_3_/316L composites through ball milling and spark plasma sintering processes. Using the Monte Carlo N-Particle Transport Code (MCNP) simulations, they established the constitutive equation of the ^155/157^Gd areal density and thermal neutron shielding rate and discovered that the thermal neutron absorption rate of Gd_2_O_3_/316L composite was ~99% when the ^155/157^Gd areal density surpassed 0.01 g/cm^2^ (Figure 8a,b). Meanwhile, Wang et al. [105] also confirmed that the thermal neutron capture rate of the Gd/316L composite was >99% when the ^155/157^Gd areal density exceeded 0.01 g/cm^2^ based on MCNP simulations. Qi et al. [106] recently undertook an analogous study to establish a mathematical link between the thermal neutron absorption rate and ^155,157^Gd areal density (A(^155,157^Gd)) (Figure 8c,d). The thermal neutron shielding performance can be directly estimated using ^155,157^Gd areal densities, which reduces the difficulty of taking numerous parameters into account. The simulation findings revealed that at a Gd concentration of 1.5 wt% and a thickness of 3 mm for such material, the thermal neutron shielding rate of Gd/316L steel was greater than 99.9%.

Theoretical modeling has greatly assisted in material design, but it has limitations. For example, recent studies by Wang et al. [105] and Qi et al. [106] revealed that the incorporation of Gd in 316L steel led to a reduction in mechanical properties and corrosion resistance with increased Gd content. This is not within the prediction range of theoretical models previously developed. Therefore, it is crucial to develop a model capable of identifying the optimal Gd concentration that can achieve a balance between neutron shielding performance, mechanical properties, and corrosion resistance.

**Figure 9:**
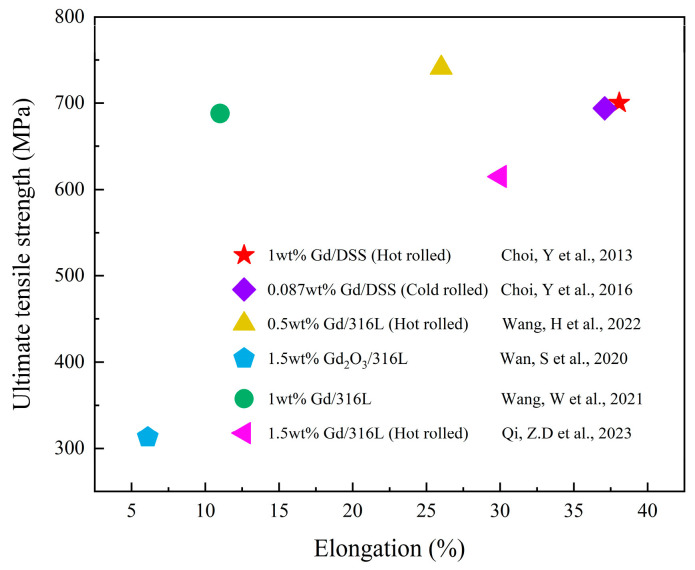
Mechanical properties of reported Gd-containing Fe-based neutron shielding materials, including: 1 wt%Gd/DSS (Hot rolled) [94], 0.087 wt%Gd/DSS (Cold rolled) [95], 0.5 wt%Gd/316L (Hot rolled) [101], 1.5 wt%Gd_2_O_3_/316L [104], 1 wt%Gd/316L [105], 1.5 wt%Gd/316L (Hot rolled) [106].

Figure 9 presents the mechanical properties of the reported Fe-based Gd-containing neutron shielding materials [94,95,101,104,105,106]. Herein, the Fe-based alloys/composites have exhibited high tensile strength and/or elongation, which are promising for use as structural-functional integrated materials.

### 4.2. Al-Based Alloys/Composites

Al alloys (e.g., Al-Mg-Si alloy) and Al matrix composites (AMCs) are widely used in the automotive and aerospace industries due to their favorable mechanical properties and lightweight nature [107]. Furthermore, they can be strengthened or modified by various particles/phases for functional purposes, such as neutron shielding materials. Among these reinforcing particles/phases, B_4_C is the most commonly used material in the AMCs due to its high strength, low density, and excellent neutron shielding properties [108]. Normally, the neutron absorption ability of the B_4_C/Al composite is highly dependent on the B_4_C content and sample thickness [109]. Chen et al. [110] discovered that the B_4_C addition enhanced the neutron shielding performance of the B_4_C/6061Al composite. However, a high proportion of B_4_C adversely affected the plasticity of the shielding composite, bringing challenges during formation. Herein, the elongation of the 30 wt%B_4_C/6061Al composite was less than 4%, making it unsuitable for use as a structural material in neutron shielding applications. The composite plasticity can be improved by decreasing the B_4_C content [111], yet this leads to a reduction in neutron shielding effectiveness at the same time. As a result, thicker shielding plates are necessary to provide the required neutron shielding performance, which contradicts the principles of our design. Furthermore, it should be noted that simply optimizing the preparation process may not be sufficient to improve composite plasticity with a high B_4_C content [20].

As a strong neutron absorber, Gd was used as a substitute for B_4_C by Xu et al. [112]. They prepared (15 wt%B_4_C + 1 wt%Gd)/Al composite by the vacuum hot pressing method, achieving a uniform distribution of both B_4_C particles and Gd phases in the Al matrix. The addition of 1 wt%Gd to 15 wt%B_4_C/Al composite resulted in an almost equivalent thermal neutron shielding performance to the 30 wt%B_4_C/Al composite. Furthermore, the (15%B_4_C + 1%Gd)/Al composite showed improved elongation (~9%) by reducing B_4_C content. The obtained results indicated that the Gd addition can substantially improve the composite plasticity while maintaining its neutron shielding performance, opening up great potential for the development of highly effective Al-based neutron shielding materials with limited neutron absorber incorporation.

**Figure 10:**
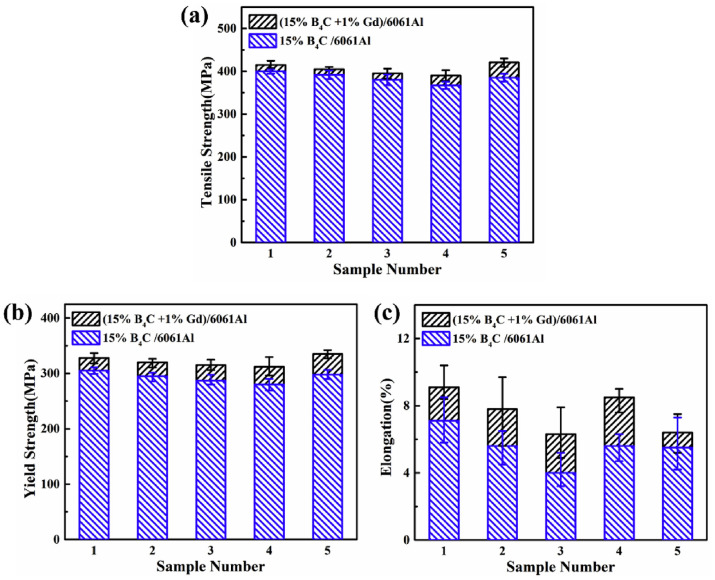
Tensile properties of the (15 wt%B_4_C + 1 wt%Gd)/Al composite and the 15 wt% B_4_C/6061Al composite prepared by hot rolling techniques [114]: (**a**) tensile strength; (**b**) yield strength; (**c**) elongation. Reproduced with permission from Xu et al., Journal of Alloys and Compounds; published by Elsevier, 2019.

In recent years, there has been growing interest in hot-working techniques for processing AMCs (e.g., hot rolling, extrusion, and forging). Hot-working approaches can improve mechanical properties by modifying the microstructure of Al composites and regulating the size and distribution of reinforcements. To improve the interface bonding and achieve a more uniform distribution of Gd phases, Xu et al. [113] subjected the as-prepared (15 wt%B_4_C + 1 wt%Gd)/Al composite to a hot extrusion process. The as-extruded (15 wt%B_4_C + 1 wt%Gd)/Al composite demonstrated an impressive 15% increase in tensile strength over its 15 wt%B_4_C/Al counterpart and showed nearly comparable levels of plasticity. The reinforced phases in the (B_4_C + Gd)/Al composite were identified as Al_2_Gd_3_, Al_5_Gd_3_O_12_, and Al_11_GdO_18_. Besides, the interaction between Gd and the Al matrix in Gd-containing AMCs leads to the formation of Al-Gd phases. Furthermore, the occurrence of an oxide layer on the original Al and Gd powders during the preparation procedure contributed to the generation of Al-Gd-O phases. Both factors are responsible for the formation of three reinforcement phases in the composite. These in situ formed phases showed higher strength and modulus than Gd particles and Al matrix, making them suitable reinforcements along with B_4_C in the composite. In their subsequent research, Xu et al. [114] performed a hot rolling process on the as-prepared (B_4_C + Gd)/6061Al composite to evaluate the effect of hot rolling on the strengthening phase, microstructure, and mechanical properties of the composite. The results revealed that the dominant Gd-containing phase was Al_5_Gd_3_O_12_ and that it did not undergo any phase transition during the hot rolling process. The size of the Gd-containing phase decreased as the amount of hot rolling deformation increased. The hot extrusion process led to a more uniform distribution of Gd-containing phases, thus enhancing the mechanical properties of the (15 wt%B_4_C + 1 wt%Gd)/6061Al composite. Here, the Gd-containing phases functioned as strengthening components to improve the tensile properties of the (15 wt%B_4_C + 1 wt%Gd)/6061Al composite in comparison with the 15 wt%B_4_C/6061Al composite (Figure 10) [114]. Additionally, the presence of stacking faults, which were the primary defects of Gd-containing phases, allowed dislocations to slip, thereby improving the plasticity of the shielding composite.

On this basis, Jiang et al. [115] carried out a more comprehensive investigation to clarify the effect of hot rolling on the mechanical properties of the (1 wt%Gd + 15 wt%B_4_C)/6061Al composite. The study concluded that the hot rolling technique resulted in a significant improvement in the mechanical properties of composites. The mechanical properties of the (Gd + B_4_C)/6061Al composite can be enhanced by two factors: (1) The hot rolling process led to a more uniform distribution of the Gd-containing phases. (2) The hot rolling process enhanced interfacial bonding strength between Gd-containing phases and the Al matrix. Besides, the thermal neutron shielding coefficient of the (1%Gd + 15%B_4_C)/6061Al composite achieved 99.9% when the sample thickness reached 3 mm. The hot rolling technique substantially improved the thermal neutron shielding performance of the composite by achieving a more uniform distribution of Gd-containing phases. Their findings provided a promising approach for the development of structural-functional integrated shielding materials.

Casting is a common method for preparing AMCs. For B_4_C/Al composite, but the high B_4_C content could impair the formability during the casting process [116]. Incorporating a smaller amount of Gd instead of B_4_C has been shown to significantly enhance the plasticity and formability of AMCs while maintaining similar neutron shielding efficiency. Nevertheless, the large-sized Gd-containing phases are prone to aggregate along grain boundaries in the casting process due to the low solubility of Gd in the Al matrix, significantly worsening the mechanical properties of the corresponding composite [117]. To avoid the problems associated with conventional casting procedures, Chen et al. [118] utilized an ultrasound-assisted casting method to fabricate (10 wt%B_4_C + 3.6 wt%Gd)/6061Al composite, followed by hot extrusion and heat treatment. The use of ultrasonic stirring has been shown to improve the uniformity of Gd-containing precipitates during solidification in the molten Al matrix. The microstructure and mechanical properties of the as-cast composite were modified using both hot extrusion and heat treatment. During this process, larger Al_3_Gd particles were broken down into smaller ones. In addition, the hot-working technique induced the precipitation of a nano-sized β phase (Mg_5_Al_2_Si_4_), which acted as a strengthening phase in the Al matrix. These modifications led to a remarkable improvement in the mechanical properties of composites. In detail, the yield strength, ultimate tensile strength, and elongation of the modified composites increased to 301 MPa (improved by 219%), 342 MPa (improved by 191%), and 2.4% (improved by 189%), respectively. However, the plasticity of this shielding composite was rather limited. Nonetheless, this study laid a solid foundation for the subsequent large-scale production of (B_4_C + Gd)/Al composites via the casting process.

The incoherent interface between B_4_C and the Al matrix is prone to delamination during the deformation process, and the low fracture toughness of B_4_C may promote the initiation and propagation of cracks. These problems greatly hinder the development of B_4_C/Al composites with higher ductility [119]. In recent years, the in situ TiB_2_/Al composite has gained considerable attention due to its good mechanical properties and high interface coherence [120]. Besides, the presence of ^10^B has made TiB_2_/Al composites a potential candidate for neutron shielding applications. Yang et al. [121] fabricated a TiB_2_/Al-Mg-Gd composite with an integrated structural-functional design using powder metallurgy methods. This composite was designed with the goal of simultaneously improving both its mechanical properties and neutron shielding performance. Structurally, the TiB_2_/Al-Mg-Gd composite exhibited high yield strength (353 ± 5 MPa), tensile strength (464 ± 6 MPa), and elongation (15.6 ± 0.4%), surpassing the available Al-based counterparts. Here, both solution strengthening by Mg atoms and grain refinement strengthening contributed significantly to the overall mechanical performance. Functionally, the TiB_2_/Al-Mg-Gd composite exhibited a high thermal neutron shielding efficiency (99%) at a limited thickness.

Pure Gd is prone to being oxidized in the air, presenting itself in the form of Gd_2_O_3_ [37]. Generally, pure Gd is relatively expensive. Therefore, Gd_2_O_3_, with its low cost and high stability, is a commonly used reinforcement for neutron shielding materials. Thus, using Gd_2_O_3_ particles instead of B_4_C particles allows for a reduction in reinforcement addition without sacrificing the shielding effectiveness or mechanical strength of the corresponding neutron shielding composite. For instance, Zhang et al. [122] fabricated a Gd_2_O_3_/6061Al composite by spark plasma sintering and hot rolling. The Monte Carlo simulation and neutron shielding tests revealed that the 10 wt%Gd_2_O_3_/6061Al composite had comparable thermal neutron shielding performance with the 30 wt%B_4_C/6061Al composite. After the ball milling process, the composite microstructure exhibited a bimodal distribution of Gd_2_O_3_ particles, where some coarse particles were reduced to the nanoscale. The primary strengthening mechanisms of composites were synergistic effects from grain boundary enhancement of Gd_2_O_3_ microparticles and grain internal enhancement of Gd_2_O_3_ nanoparticles. The fracture mechanism observed in the 10 wt%Gd_2_O_3_/6061Al composite was a ductile fracture. Later, Li et al. [123] investigated the microstructure evolution and strengthening mechanisms of the hot-rolled Gd_2_O_3_/6061Al composite. As the deformation progressed, Gd_2_O_3_ particles were found dispersed evenly throughout the 6061Al matrix. It was confirmed that the Gd_2_O_3_ particles played a crucial role in promoting dynamic recrystallization nucleation while simultaneously inhibiting grain growth. The strengthening mechanism of the Gd_2_O_3_/6061Al composite was primarily attributed to Orowan strengthening, and the related fracture mechanisms shifted from brittle to ductile after hot rolling. Similarly, Cong et al. [124] designed and fabricated a Gd_2_O_3_/6063Al composite, which exhibited better thermal neutron shielding efficiency than the 30 wt%B_4_C/Al composite.

The neutron absorption process is obtained through the Gd(n, γ) reaction. The secondary γ-ray, a by-product of the Gd(n, γ) reaction, poses a great threat to the surroundings. Furthermore, the occurrence of high-energy neutron and γ-ray radiation is simultaneous in realistic nuclear radiation. However, the current Gd-containing neutron shielding materials are still inadequate for γ-ray radiation shielding. Thus, the design of Gd-containing neutron shielding materials may include effective γ-ray radiation protection as well.

**Figure 11:**
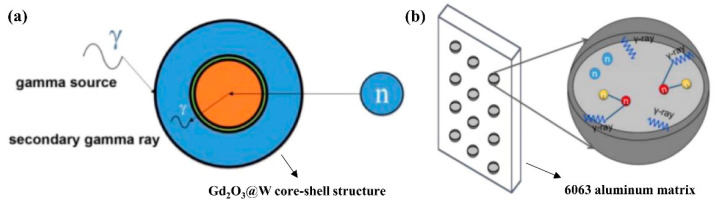
Gd_2_O_3_@W core–shell structure for double shielding against γ-ray and neutron radiation [127]: (**a**) schematic diagram of Gd_2_O_3_@W core–shell structure particles; (**b**) 6063 aluminum composite plate doped with Gd_2_O_3_@W core–shell structure particles. Reproduced with permission from Zhang et al., Materials Letters; published by Elsevier, 2020.

Tungsten (W) is frequently employed as a reinforcing phase in radiation shielding composites considering its outstanding performance as γ-ray shielding components [125]. To overcome the limitations of current neutron shielding materials employed in the storage of γ-ray radiation protection, Cong et al. [126] designed a core–shell structure with a W outer layer and a Gd_2_O_3_ inner layer, and 6063Al was used as the matrix for structure support. They found that *x*W@25wt%Gd_2_O_3_/6063Al (*x* = 7, 15, 25, and 35 wt%) composite showed increased radiation shielding ability for both thermal neutrons (<0.15 eV) and γ-rays compared with their commercial 30 wt%B_4_C/Al counterparts. In-depth investigations have revealed that an extended sintering time and higher sintering temperature, coupled with an elevated W content, contributed to a substantial increase in the microhardness of the W@25wt%Gd_2_O_3_/6063Al composite. Similarly, Zhang et al. [127] fabricated the Al composite reinforced by particles consisting of micron-sized Gd_2_O_3_ cores and nano-sized W shells (Figure 11). In Figure 11, the outer W shell of Gd_2_O_3_@W composite can effectively protect against both secondary γ-rays in the shielding process and γ-rays in the radiation environment, filling the gap of low efficiency of Gd_2_O_3_/Al composite in γ-ray radiation shielding. Monte Carlo simulations demonstrated that the neutron shielding ability and the half-value layers for primary and secondary γ-rays met the necessary criteria for effective neutron shielding applications. The core–shell structure particles led to an improvement in both tensile strength and elongation of the shielding composite compared with the contrast sample. Both fine-grain strengthening and Orowan strengthening made significant contributions to the mechanical strength of the shielding composite. Notably, the tensile fracture morphology revealed that the trans-granular fracture of the composite was caused predominantly by the presence of grain-boundary Gd_2_O_3_@W particles, which hindered crack propagation.

It is widely acknowledged that Gd is an effective absorber of thermal neutrons, but its shielding effectiveness against fast neutron and γ-ray radiation is limited. Therefore, additional shielding components are necessary to design materials with multi-functional shielding applications. Recent studies by Cong et al. [126] and Zhang et al. [127] have demonstrated the potential of core–shell structures in shielding materials. Currently, the manufacturing of neutron and γ-ray co-shielding materials involves separate production and subsequent combination for practical use. Integrating different components into a functional unity and improving the connection stability between various components are crucial.

**Figure 12:**
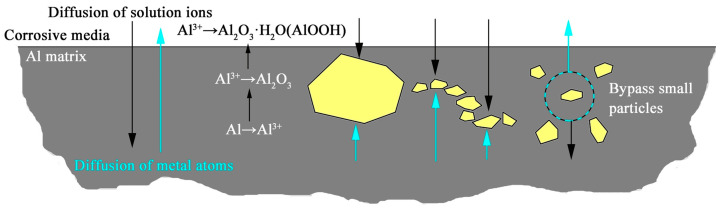
Schematic of the corrosion mechanism of Al-based Gd_2_O_3_-W shielding composites with different particle distributions [128]. Reproduced with permission from Cong et al., Corrosion Science; published by Elsevier, 2023.

Pitting corrosion, which is the primary form of corrosion for Al-based composites used for the storage of SNFs, can be inhibited by Gd-containing particles (i.e., Al_3_Gd) in the W@Gd_2_O_3_/Al composite [128]. However, this study also revealed that Fe^+^ irradiation (used to simulate neutrons and γ-ray irradiation) led to the formation of numerous cavities on the surface layer of the composite, and these defects provided fast diffusion paths for corrosive ions, resulting in an accelerated corrosion rate. Therefore, resistance to corrosion and radiation damage should also be considered to ensure the reliability of shielding materials. Herein, Figure 12 summarizes the influence of particle size and distribution on the corrosion behavior of shielding composites [128]. The Gd-containing particles were coarsened significantly in the sample sintered at high temperatures, leaving large pure-Al regions exposed and leading to localized corrosion. During the corrosion process, the corrosive media diffused inward, and the concentration of corrosive ions decreased with depth. In addition, metallic Al was first oxidized to Al^3+^, and then hydrolyzed to form Al_2_O_3_ and AlO(OH) oxide layers.

Table 3 lists the reported Gd-containing AMCs designed for thermal neutron shielding. Herein, Gd was first used as a substitute for B_4_C in Al-based shielding materials [112,113,114,115]. Researchers found that adding extremely tiny quantities of Gd may effectively improve the neutron absorption ability of shielding composites, addressing the problem of poor plasticity in B_4_C/Al composites by replacing Gd for B while maintaining the same level of neutron shielding performance. Thereafter, the more cost-effective and stable Gd_2_O_3_ ceramic particle was proposed as an alternative neutron absorber in Al-based shielding materials [122,124]. Later, to fill the gap of Gd_2_O_3_ in both fast neutron attenuation and γ-ray shielding fields, a core–shell structure with the W shell and the Gd_2_O_3_ core was designed [126,127]. Gd_2_O_3_ particles can effectively shield the incident neutrons and γ-rays, improving the overall shielding performance of AMCs. However, there is a compromise between the mechanical properties and neutron shielding performance, and fabricating a structural material with high neutron shielding efficiency is an important area for future study.

### 4.3. Other Metal-Based Alloys

In the 2000s, many in-depth studies on Ni-Cr-Mo-Gd alloys for neutron shielding materials were conducted in the United States for the Yucca Mountain Project [129]. Initially, Dupont et al. [130] investigated the effects of Gd on the solidification behavior and weldability of a Ni-Cr-Mo alloy and discovered that the Ni-Cr-Mo-Gd alloy solidified in a way comparable to a binary eutectic system. The solidification of Ni-Cr-Mo-Gd alloy began with a primary L → γ reaction and ended with a eutectic type L → γ + Ni_5_Gd reaction at 1258 °C. In addition, the sensitivity to solidification cracking of Ni-Cr-Mo-Gd alloy was highest with 1 wt%Gd and declined with further Gd additions. Mizia et al. [131] found that the strength levels of Ni-Cr-Mo-Gd alloy were 10% higher in both longitudinal and transverse orientations than the base (Gd-free) Ni-Cr-Mo alloy. The corrosion resistance of Ni-Cr-Mo-Gd alloy was discovered to be related to the Gd amount, which determined the quantity of gadolinide (Ni_5_Gd) presented in the alloy. Likewise, Lister et al. [132] evaluated the corrosion resistance of Ni-Cr-Mo-Gd alloys. In electrochemical and longer-term immersion tests, the Ni_5_Gd phase was stable in both conventional and simulated test conditions. Nevertheless, further tests in more corrosive environments and at higher temperatures are required to fully ascertain its corrosion resistance and thermal stability.

However, the high Mo content in Ni-Cr-Mo-Gd alloys poses a limitation to their promotion and application considering their high manufacturing costs. Therefore, Zhang et al. [133] proposed a Ni-Cr-Fe-Gd alloy (i.e., Modified-690Gd), and this alloy was composed primarily of a γ-austenite matrix with a (Ni, Cr, Fe)_5_Gd phase featuring hexagonal crystal structure along grain boundaries. The (Ni, Cr, Fe)_5_Gd phase content increased with higher Gd concentration, resulting in reduced fracture elongation of the alloy. Despite this, the Modified-690Gd alloy showed good tensile strength exceeding 620 MPa and a minimum elongation of 50%, indicating excellent mechanical properties. Additionally, the Ni-Cr-Fe-Gd alloy displayed favorable corrosion resistance, and the (Ni, Cr, Fe)_5_Gd phase within the alloy was preferentially attacked and removed during the corrosion process. When the Gd content in the alloy is 2.0 wt% and the thickness exceeds 2 mm, the neutron transmittance can be significantly reduced to values below 0.01. It is worth noting that when subjected to identical neutron shielding test conditions, the Modified-690Gd alloy demonstrated a lower thickness in comparison with B-containing alloys, providing size advantages as neutron shielding components.

Based on the outstanding mechanical properties and corrosion resistance of Ti, Hur et al. [134] developed a novel Ti-Gd alloy with varying Gd contents. Herein, the Gd-rich phases were discovered randomly dispersed inside the Ti matrix, and the average chemical composition of the particles was 95.96 wt%Gd, 3.71 wt%Ti, and 0.32 wt%O. In the rolling direction, the elongated phases showed no signs of brittle fracture or gaps between the phases and the Ti matrix, suggesting excellent deformability of Gd-rich phases. In the simulated SNF pool water containing boric acid at 50 °C, the corrosion tests found that Ti-Gd alloy had lower corrosion potentials, higher corrosion current density values, and higher passive current density values than pure Ti. This study provided valuable information on the corrosion behavior of Ti-Gd alloy in wet storage. Unfortunately, the mechanical properties and neutron shielding performance of this alloy were not evaluated there.

## 5. Summary

Gd has outstanding neutron shielding ability, primarily against thermal neutrons, and it has been widely used as a neutron absorber in inorganic nonmetallic-based, polymer-based, and metallic-based shielding materials. In inorganic nonmetallic neutron shielding materials, the major Gd-containing form is Gd_2_O_3_. Among polymer neutron shielding materials, Gd can exist in the form of Gd-related structures (i.e., Gd-MOF and Gd@MXene). Furthermore, Gd is found in metal shielding materials as Gd-containing phases (e.g., (Fe, Ni, Cr)_3_Gd, Ni_5_Gd, and Al_5_Gd_3_O_12_).

Depending on the features of the matrix, different types of materials can be applied to various shielding scenarios. Inorganic nonmetallic materials are frequently employed in the building and construction industries. In contrast, given the lightweight and flexible nature of polymers, they are more suitable for use as wearable protective devices. Metallic materials are preferred for storing and transporting SNFs due to their high thermal stability, mechanical strength, and corrosion resistance. The incorporation of Gd substantially improves the neutron shielding performance of these materials, allowing for enhanced neutron shielding efficiency at reduced thickness and restricted geometrical size. After the review of the available reports on the novel Gd-containing shielding materials, there are still several challenges remaining in this area:

1. There is a mutually restrictive relationship between neutron shielding performance and the mechanical properties of Gd-containing shielding materials. Increasing the Gd content has the potential to improve the thermal neutron absorbing abilities of shielding materials, but excessive Gd content may lead to a reduction in mechanical properties (e.g., plasticity and strength). Furthermore, the high quantities of Gd may complicate the manufacture of shielding materials, e.g., the powder metallurgy method plus the deformation technique for metal-based materials. Therefore, the manipulations of the preparation procedures, neutron shielding performance, and mechanical properties are tough but critical. Possibly, a potential research direction is to establish theoretical models capable of determining the appropriate Gd addition for the controllable fabrication process, thus obtaining a trade-off between neutron shielding performance and mechanical properties.
2. Although Gd possesses a high thermal neutron absorbing ability, its shielding efficiency against fast neutrons is negligible. Moreover, the absorption of neutrons by Gd is accompanied by the release of secondary γ-rays, thereby posing a potential radiation hazard. In other words, both neutron and γ-ray radiation may occur simultaneously in practical applications. Therefore, shielding materials must have effective neutron shielding abilities for both fast and thermal neutrons, as well as adequate γ-ray radiation shielding performance. H-rich materials (e.g., polymers) and concretes are considered to be the optimal choice for attenuating fast neutrons. Thus, they can be used as the matrix to improve the overall neutron shielding performance of Gd-containing shielding material. For the deficiency of Gd-containing materials in shielding γ-ray radiation, incorporating components with high γ-ray radiation shielding properties (e.g., W, concrete, steel) is feasible. For instance, the Gd_2_O_3_@W core–shell structure with a W outer layer has proven to be promising for both neutron and γ-ray radiation shielding.
3. It is still challenging to improve the neutron shielding performance of Gd-containing materials. In this regard, regulating the form of Gd in the matrix and ensuring a homogenous distribution of Gd components is critical in the fabrication of Gd-containing shielding materials. Specifically, it is important to modify the preparation process of shielding materials to improve the homogeneity of the distribution of Gd-containing phases, promote the generation of reinforcing phases that are beneficial to the material properties, and avoid the appearance of harmful secondary phases. In addition, there are some innovative material preparation technologies, e.g., additive manufacturing, that allow for the fabrication of custom-designed samples with more freedom. This novel material processing method may give more options for meeting the demands of practical applications.

Currently, there are some problems in the development and application of Gd-containing shielding materials. Besides, it is noteworthy that the cost of metallic Gd remains relatively expensive in comparison to other neutron absorbers (e.g., B_4_C). Despite this, there is no doubt that the novel Gd-containing materials have broad application prospects as neutron shielding materials. We hope that our work will inspire and guide further research in this field.

## Figures and Tables

**Table 1:** Neutron capture cross-sections of several typical neutron absorbing elements [13].

Element	Corresponding Isotope	Atomic Number	Relative Atomic Mass	Neutron Capture Cross-Sections/Barns	Abundance/wt%
Boron	^10^B	5	10.81	3840	19.9
Cadmium	^113^Cd	48	112.41	20,600	12.22
Samarium	^149^Sm	62	150.36	40,000	13.8
Gadolinium	^155^Gd	64	157.25	62,540	14.8
	^157^Gd			255,000	15.65

**Table 2:** Neutron shielding performance of reported Gd-containing PMCs.

Year	Matrix	Chemical Composition	Content of Gd-Containing Components	Neutron Shielding Performance *	Main Features	Ref.
2018	PMMA	poly(MMA-coGd(MAA)_3_)	1.91 wt%	I/I_0_ = 0.1 (Sample thickness: 5 mm)	Self-polymerization	[70]
2018	HDPE	*h*-BN/Gd_2_O_3_/HDPE	3 wt% (with 7 wt%BN)	I/I_0_ ≈ 0.3 (Sample thickness: 5 cm)	Hybridization of *h*-BN and Gd_2_O_3_	[82]
2019	PAN	Gd(MAA)_3_/PAN	12.11 wt%	I/I_0_ = 0.01 (Sample thickness: 2 mm, by simulation)	Self-polymerization	[71]
2019	EP	Gd-MOF/EP	10 wt%	I/I_0_ < 0.1 (Sample thickness: 5 cm, by simulation)	MOF-based	[78]
2020	PVA	Gd_2_O_3_/PVA	3.5 wt%	I/I_0_ = 0.43 (Sample thickness: 5 mm, by simulation)	Self-polymerization	[69]
2021	PI	Gd-MOF/PI	3 wt%	I/I_0_ = 0.096 (Sample thickness: 5 cm, for fast neutrons, by simulation)	MOF-based	[79]
				I/I_0_ = 0.009 (Sample thickness: 0.2 cm, for thermal neutrons, by simulation)		
2021	HDPE	Gd_2_O_3_/B_4_C/HDPE	10 wt% (with 10 wt% B_4_C and 80 wt% HDPE)	I/I_0_ = 0.1 (Sample thickness: 9.1 cm)	Surface modification; nanoparticles	[86]
2022	NR	Gd_2_O_3_/NR	50 phr	I/I_0_ = 0.55 (Sample thickness: 2 mm)	Self-healing ability	[74]
2022	PVA	Gd@MXene/PVA	15.1 wt%	I/I_0_ = 0.169 (Sample thickness: 1 mm)	MXene-based	[76]
2022	SAPPS	Gd_2_O_3_/SAPPS	10 wt%	I/I_0_ = 0.2 (Sample thickness: 2 cm)	Nanoparticles	[85]

* Here, the average counts of neutrons detected with and without specimens are noted as I and I_0_, respectively. I/I_0_ refers to the neutron transmission ratio, and the neutron shielding efficiency is higher in materials with a lower I/I_0_ ratio.

**Table 3:** Several Gd-containing AMCs developed for neutron shielding application.

Year	Matrix	Chemical Composition *^,1^	Mechanical Properties	Thermal Neutron Shielding Performance *^,2^	Ref.
2016	Pure Al	(1%Gd + 15%B_4_C)/Al	UTS = 170 MPa, EL ≈ 9%	I/I_0_ ≈ 0 (sample thickness: 3 mm)	[112]
2017	Pure Al	(1%Gd + 15%B_4_C)/Al	UTS = 260 MPa, EL ≈ 7%	Not mentioned	[113]
2019	6061Al	(1%Gd + 15%B_4_C)/6061Al	UTS = 421 MPa, EL ≈ 6.4%	Not mentioned	[114]
2019	6061Al	(1%Gd + 15%B_4_C)/6061Al	UTS = 380 MPa, EL = 5%	I/I_0_ ≈ 0 (sample thickness: 3 mm)	[115]
2019	6061Al	10%Gd_2_O_3_/6061Al	UTS = 240 MPa, EL = 16%	I/I_0_ = 0.0036 (sample thickness: 10 mm)	[122]
2020	6063Al	25%Gd_2_O_3_/Al	Microhardness = 120 HV_0.1_	I/I_0_ ≈ 0 (sample thickness: 0.75 mm)	[124]
2020	Pure Al	5%Gd_2_O_3_@20%W/Al	UTS = 310 MPa, EL ≈ 3%	I/I_0_ = 0.01 (sample thickness: 3 mm, by simulation)	[127]
2022	6061Al	(10%B_4_C + 3.6%Gd)/6061Al	UTS = 342 MPa, EL = 2.4%	I/I_0_ = 0.002 (sample thickness: 3 mm, by simulation)	[118]
2022	AlMg	1%Gd/6%TiB_2_/Al–6%Mg	UTS = 464 MPa, EL = 15.6%	I/I_0_ ≈ 0 (sample thickness: 8 mm)	[121]

*^,1^ All units in the chemical composition of shielding materials are provided in weight percent (wt%). *^,2^ Similarly, the average counts of neutrons detected with and without specimens are noted as I and I_0_, respectively. I/I_0_ refers to the neutron transmission ratio, and the neutron shielding efficiency is higher in materials with a lower I/I_0_ ratio.

## Data Availability

Not applicable.
